# Allosteric enhancement of the BCR-Abl1 kinase inhibition activity of nilotinib by cobinding of asciminib

**DOI:** 10.1016/j.jbc.2022.102238

**Published:** 2022-07-06

**Authors:** Baswanth Oruganti, Erik Lindahl, Jingmei Yang, Wahid Amiri, Rezwan Rahimullah, Ran Friedman

**Affiliations:** Department of Chemistry and Biomedical Sciences, Faculty of Health and Life Sciences, Linnæus University, Kalmar, Sweden

**Keywords:** BCR-Abl1 kinase, tyrosine kinase inhibitors, allosteric inhibition, drug resistance, combination therapy, ATP, adenosine triphosphate, Abl1, Abelson tyrosine kinase, BCR, breakpoint cluster region, CML, chronic myeloid leukemia, CI, combination index, D–A, donor-acceptor, DFT, density functional theory, MD, molecular dynamics, nH, nonhydrogen, NCI, noncovalent interaction, PDB, Protein data bank, RMSD, root mean squared deviation, TKIs, tyrosine kinase inhibitors, wT-metaD, well-tempered metadynamics, WT, wildtype

## Abstract

Inhibitors that bind competitively to the ATP binding pocket in the kinase domain of the oncogenic fusion protein BCR–Abl1 are used successfully in targeted therapy of chronic myeloid leukemia (CML). Such inhibitors provided the first proof of concept that kinase inhibition can succeed in a clinical setting. However, emergence of drug resistance and dose-dependent toxicities limit the effectiveness of these drugs. Therefore, treatment with a combination of drugs without overlapping resistance mechanisms appears to be an appropriate strategy. In the present work, we explore the effectiveness of combination therapies of the recently developed allosteric inhibitor asciminib with the ATP-competitive inhibitors nilotinib and dasatinib in inhibiting the BCR–Abl1 kinase activity in CML cell lines. Through these experiments, we demonstrate that asciminib significantly enhances the inhibition activity of nilotinib, but not of dasatinib. Exploring molecular mechanisms for such allosteric enhancement *via* systematic computational investigation incorporating molecular dynamics, metadynamics simulations, and density functional theory calculations, we found two distinct contributions. First, binding of asciminib triggers conformational changes in the inactive state of the protein, thereby making the activation process less favorable by ∼4 kcal/mol. Second, the binding of asciminib decreases the binding free energies of nilotinib by ∼3 and ∼7 kcal/mol for the wildtype and T315I-mutated protein, respectively, suggesting the possibility of reducing nilotinib dosage and lowering risk of developing resistance in the treatment of CML.

Reducing dose-limiting off-target effects and preventing emergence of drug resistance are two key challenges in targeted therapy of cancer. In cancers such as chronic myeloid leukemia (CML) and several acute lymphocytic leukemias, Abelson tyrosine kinase (Abl1) protein becomes constitutively active due to genetic fusion of the Abl1 gene with the breakpoint cluster region (BCR) gene which is transcribed and translated to the BCR-Abl1 fusion protein. The fusion protein lacks N-cap for myristoylation that plays a central role in negative regulation of activity of the wildtype (WT) Abl1 kinase. Imatinib was the first drug to be discovered in the targeted therapy treatment of CML and to date remains one of the most successful drugs in its first-line treatment (1). It inhibits the activity of the BCR-Abl1 fusion protein by binding competitively to the ATP-binding site in the kinase domain of the inactive state of the protein (see Fig. 1). However, widespread use of imatinib in clinical settings resulted in the development of point mutations in the kinase domain leading to emergence of resistance against it (2).

**Figure 1:**
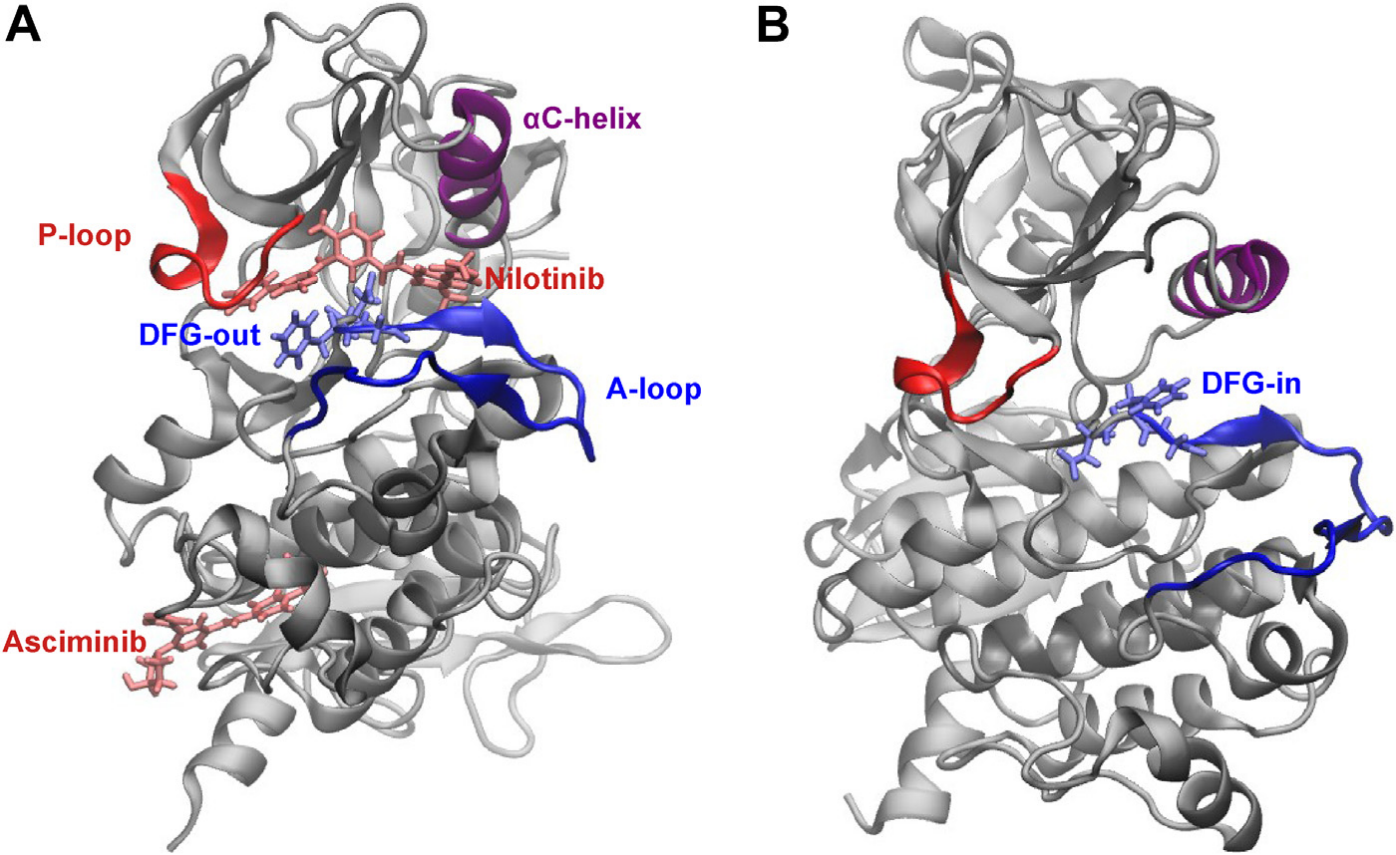
**Structures of the inactive state bound to asciminib and nilotinib, and the active state of the Abl1 kinase.** Note the four key structural elements, namely the A-loop, the αC-helix, the DFG motif, and the P-loop, and the difference in DFG conformation between the inactive DFG-out (***A***) and active DFG-in (***B***) states. Abl1, Abelson tyrosine kinase.

A second-generation of tyrosine kinase inhibitors (TKIs), namely, nilotinib (3), dasatinib (4), and bosutinib (5, 6), were developed to address this problem. While these inhibitors also act in a manner similar to that of imatinib in terms of their orthosteric inhibition of ATP binding, dasatinib binds to the active state whereas the other two bind the inactive state (7, 8). Although the second-generation drugs effectively neutralize many of the imatinib-resistant mutants (besides inhibiting the WT BCR-Abl1), they fail against the T315I gatekeeper mutant (9). A third generation TKI called ponatinib, that also binds in the kinase domain of the inactive state of the protein, was subsequently developed to inhibit even the T315I mutant (10). However, serious adverse effects were reported with ponatinib (11), which limited its clinical application to a last resort treatment option after failure of two or more TKIs. Furthermore, emergence of compound mutations, *i.e.*, two mutations in the same copy of the gene, such as Y253H/E255V, G250E/T315I, Y253H/T315I, and E255V/T315I were identified that can cause resistance even against ponatinib treatment (12) as these mutations make the protein more active (13).

Recently, asciminib, an allosteric inhibitor, that binds to the myristate pocket in a regulatory domain of the inactive state of the protein (14) was approved by the Food and Drugs Administration to treat patients who previously failed two or more TKIs or patients diagnosed with the T315I mutation. The binding of asciminib restores the lost negative regulatory activity of the myristate pocket of Abl1 and locks its conformation in the inactive state, thus inhibiting the BCR-Abl1 activity (14, 15). A distinct advantage of asciminib is that it retains substantial activity against many point mutations in the kinase domain which confer resistance against ATP-competitive TKIs as its binding site is remote from the ATP-binding site of the kinase domain as shown in Figure 1 (16). Nevertheless, resistance can still emerge against asciminib due to mutations in the myristate pocket as well as in the linker region (16). Moreover, T315I-inclusive compound mutations in the kinase domain can also cause resistance against asciminib (17). While the mutations in the myristate pocket sterically impede the drug binding, the latter two types of mutations may confer resistance indirectly by shifting the equilibrium toward the active state or by increasing the catalytic turnover rate (16). Alternatively, resistance against asciminib (as well as against other TKIs) can also emerge by Abl1-independent mechanisms, for example, by upregulation of ABCG2 transporter protein that causes increased drug efflux (18).

To address the problem of multidrug resistance in the treatment of CML, it is desirable to develop treatment protocols that reduce the emergence of drug resistance. In this regard, it was reported that combining asciminib with the ATP-competitive inhibitors nilotinib (14, 17) or ponatinib (17) enhances Abl1 kinase inhibition and suppresses the emergence of resistance in Ph+ leukemia cells isolated from blood of CML patients (17) as well as in KCL-22 mouse xenograft models (14). Furthermore, inclusion of asciminib significantly reduced the concentration of ponatinib required to inhibit T315I-including compound mutants (17, 19). In this light, we have previously explored the effects of combination therapy of asciminib with imatinib or dasatinib on KCL-22 cells and observed synergism between the drugs only at high degrees of inhibition (20). A recent clinical experience showed that a patient diagnosed with chronic phase CML and previously failed monotherapy of all approved TKIs (except ponatinib which was not used) due to emergence of the F317L mutation responded to combination therapy treatment of asciminib with bosutinib and achieved a major molecular response (21). In view of these encouraging findings on the beneficial effects of combination therapies, the present study aims to further explore the merits or limitations of the combination therapies of asciminib with nilotinib or dasatinib by estimating the differences in the IC_50_ values of these drugs between the monotherapy and combination therapy in K562 and KCL-22 cells. To the best of our knowledge, these differences were not previously reported.

Based on previous molecular dynamics (MD) simulations on Abl1 kinase (22, 23), it can be hypothesized that one reason for success of the combination therapies might be allosteric communication between the myristate and the ATP-binding pockets. However, detailed molecular mechanisms underlying the efficacy or inefficacy of the combination therapies remain unexplored to date. The present work aims to fill in this important gap by computationally investigating molecular mechanisms governing drug synergism in the combination therapies.

First, by performing cell line experiments, we explored any potential benefits of combination therapies of asciminib+nilotinib and asciminib+dasatinib in inhibiting BCR-Abl1 kinase activity over corresponding monotherapies of asciminib, nilotinib, and dasatinib. As nilotinib and dasatinib bind different Abl1 conformations (inactive *versus* active) (7), it was also of interest to test whether asciminib has any differential influence on the activities of the two drugs. Indeed, having found that co-binding of asciminib modulates the IC_50_ of nilotinib but not that of dasatinib, we then investigated molecular mechanisms underlying the modulation by a detailed computational study using MD, metadynamics, and quantum chemical calculations.

## Results and discussion

In the following, we first present a comparison of the dose–response curves for monotherapies of asciminib, nilotinib, and dasatinib with those for combination therapies of asciminib+nilotinib and asciminib+dasatinib in cell line experiments. Subsequently, we explored how the binding of asciminib influenced the activation dynamics of the Abl1 kinase by performing MD and metadynamics simulations. Finally, we investigated whether the binding affinity of nilotinib is altered by the presence of asciminib by carrying out quantum chemical calculations on small representative models of the Abl1–nilotinib complex generated from MD simulations of the asciminib+nilotinib bound and nilotinib-only bound Abl1 complexes.

### Cell line experiments reveal increased BCR-Abl1 inhibitory activity of nilotinib in the presence of asciminib

**Figure 2:**
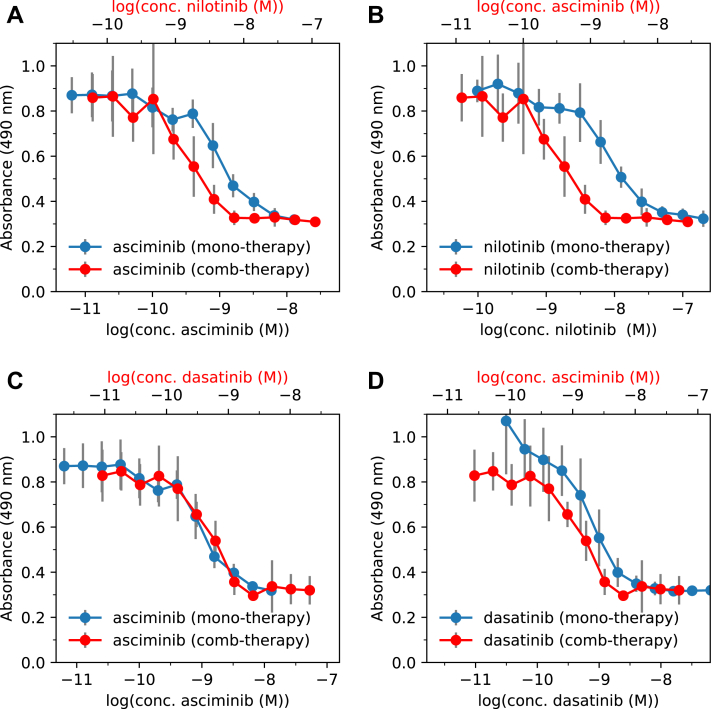
**Dose–response curves comparing combination therapies and monotherapies of different drugs.** Panels ***A*** and ***B*** show results from asciminib+nilotinib combination therapy and the corresponding monotherapies. Panels ***C*** and ***D*** display results from asciminib+dasatinib combination therapy and the corresponding monotherapies. Standard deviations of absorbances are shown as *gray vertical bars*. Note that the second X-axis marked in *red applies* only for the combination therapy.

**Figure 3:**
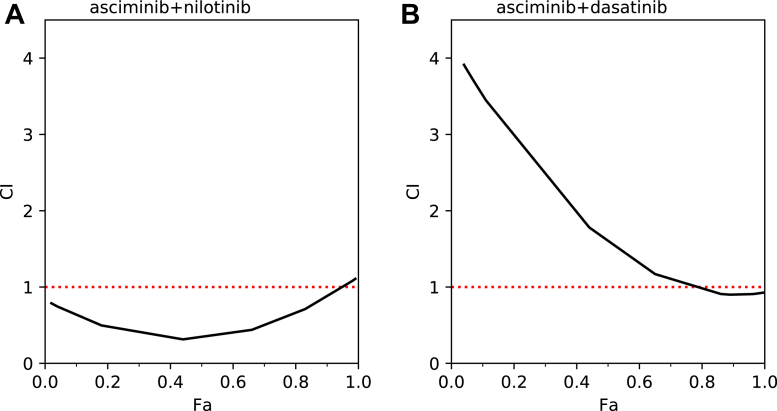
**Combination index (CI) values plotted as a function of fraction of affected cells (Fa) in K562 cell lines.** Panels ***A*** and ***B*** correspond to asciminib+nilotinib and asciminib+dasatinib combination therapies, respectively. CI < 1 indicates synergism, and CI >1 indicates antagonism.

The dose–response curves for combination therapies of asciminib+nilotinib and asciminib+dasatinib were measured in K562 cells and compared with those for monotherapies of asciminib, nilotinib, and dasatinib (see Fig. 2). The associated IC_50_ values of the drugs are presented in Table 1. It is notable from Figure 2 that the growth of BCR-Abl1 positive cells is strongly inhibited by all the three drugs in both monotherapy and in combination therapy. The monotherapy IC_50_ values of 0.9, 7.0, and 0.5 nM, respectively, for asciminib, nilotinib, and dasatinib are in accordance with the previously reported IC_50_ ranges of 0.2 to 8.0 (14, 15, 16, 17), 3 to 30 (3, 24, 25, 26, 27), and 0.2 to 1.0 (28, 29, 30, 31) nM for these drugs. Furthermore, it can be observed from Figure 2 (panels *A* and *B*) that the efficacy of both asciminib and nilotinib are enhanced in the asciminib+nilotinib combination therapy relative to those in the monotherapies of these drugs. This enhancement is particularly prominent for nilotinib, wherein relatively larger variations in cell viabilities are observed between the combination therapy and monotherapy at the same drug concentrations (Fig. 2*B*). Additionally, combination index (CI) plots presented in Figure 3*A* reveal synergistic interactions (CI < 1) between asciminib and nilotinib. Accordingly, the IC_50_ value for nilotinib is ∼5 times smaller in the combination therapy compared to that in the monotherapy (Table 1). To further ascertain these findings and to test the effectiveness of the asciminib+nilotinib combination therapy in cells that develop nilotinib resistance, cell line experiments were also performed using KCL-22 cells and nilotinib-resistant KCL-22 (referred to as KCL-22NR) cells. The dose–response curves obtained from these experiments are shown in Fig. S1 of the Supporting Information, and the associated IC_50_ values of the drugs in monotherapy and combination therapy are provided in Table 1.

**Table 1:** IC_50_ (nM) values for mono and combination (comb) therapies of different inhibitors (I1 and I2)

Cell line	I1	Therapy	I2	IC_50_^I1^	IC_50_^I2^
K562	Asciminib	Mono	None	0.9	–
	Nilotinib	Mono	None	7.0	–
	Dasatinib	Mono	None	0.5	–
	Asciminib	Comb	Nilotinib	0.3	1.5
	Asciminib	Comb	Dasatinib	1.2	0.5
KCL-22	Asciminib	Mono	None	0.3	–
	Nilotinib	Mono	None	9.4	–
	Asciminib	Comb	Nilotinib	–b	2.3
KCL-22NR^a^	Asciminib	Mono	None	0.9	–
	Nilotinib	Mono	None	14.6	–
	Asciminib	Comb	Nilotinib	–b	6.9

a A nilotinib-resistant KCL-22 cell line. These cells showed much increased growth when cultured in the presence of 10 nM nilotinib (Fig. S2 of the SI).

b Concentration of asciminib in the combination therapy kept fixed at its IC50 value of 0.3 nM in monotherapy against KCL-22 cells.

In accordance with the results from K562 cells, the IC_50_ value for nilotinib is ∼4 times lower in the combination therapy compared to that in the monotherapy in KCL-22 cells (Table 1). Based on these *in vitro* findings in different cell lines, it can be predicted that it is possible to lower the dosage of nilotinib substantially in treatment of CML while maintaining the same response effect by means of its combination therapy with asciminib, which is consistent with recent findings on asciminib+ponatinib combination therapy (17, 19). Reduced dosages, in turn, result in lower plasma drug concentrations and a concomitant reduction in dose-dependent adverse effects of nilotinib or a higher efficacy with normal doses. Furthermore, it is clear from Table 1 that nilotinib when combined with asciminib is about two times more effective than nilotinib monotherapy (see Fig. S2 of the SI for results on emergence of resistance against nilotinib monotherapy) against KCL-22NR cells, although further studies are required to characterize specific resistance mutations that emerged. This finding is in line with the recent reports that combination of asciminib with nilotinib prevented emergence of drug resistance in KCL-22 mouse xenograft models (14) and Ba/F3 BCR-Abl1 cells (17).

Turning to the asciminib+dasatinib combination therapy, the inhibitory activities of asciminib and dasatinib varied little between monotherapies and combination therapy as can be seen from Figure 2 (panels *C* and *D),* and from the similar IC_50_ values of the drugs in both therapies (0.9 *versus* 1.2 nM for asciminib, 0.5 *versus* 0.5 nM for dasatinib, see Table 1). However, the CI plots in Figure 3*B* show antagonism (CI > 1) between the drugs for fraction of affected cells (Fa) below 80%. This result complements our recent finding that asciminib synergizes with dasatinib in KCL-22 cells only for very high Fa values (>80%) (20). Thus, asciminib+dasatinib combination therapy appears far less effective than asciminib+nilotinib combination therapy. One possible reason for contrasting results between asciminib+nilotinib and asciminib+dasatinib combination therapies might be differences in how the binding of asciminib affects that of nilotinib and dasatinib. Particularly, it is interesting to note that while nilotinib binds to the inactive (DFG-out) state of the protein, dasatinib on the other hand binds to the active (DFG-in) state (7). Therefore, it is plausible that some kind of allosteric communication exists between asciminib and nilotinib which is not possible between asciminib and dasatinib.

To unveil molecular mechanisms behind allosteric interactions between asciminib and nilotinib, we performed a thorough computational investigation, which we turn to next, on how the binding of asciminib influences the activation dynamics of Abl1 kinase and the binding affinity of nilotinib by carrying out MD, metadynamics simulations, and quantum chemical calculations.

### MD and metadynamics simulations show changes in conformational dynamics of the inactive state upon the binding of asciminib

#### MD simulations

**Figure 4:**
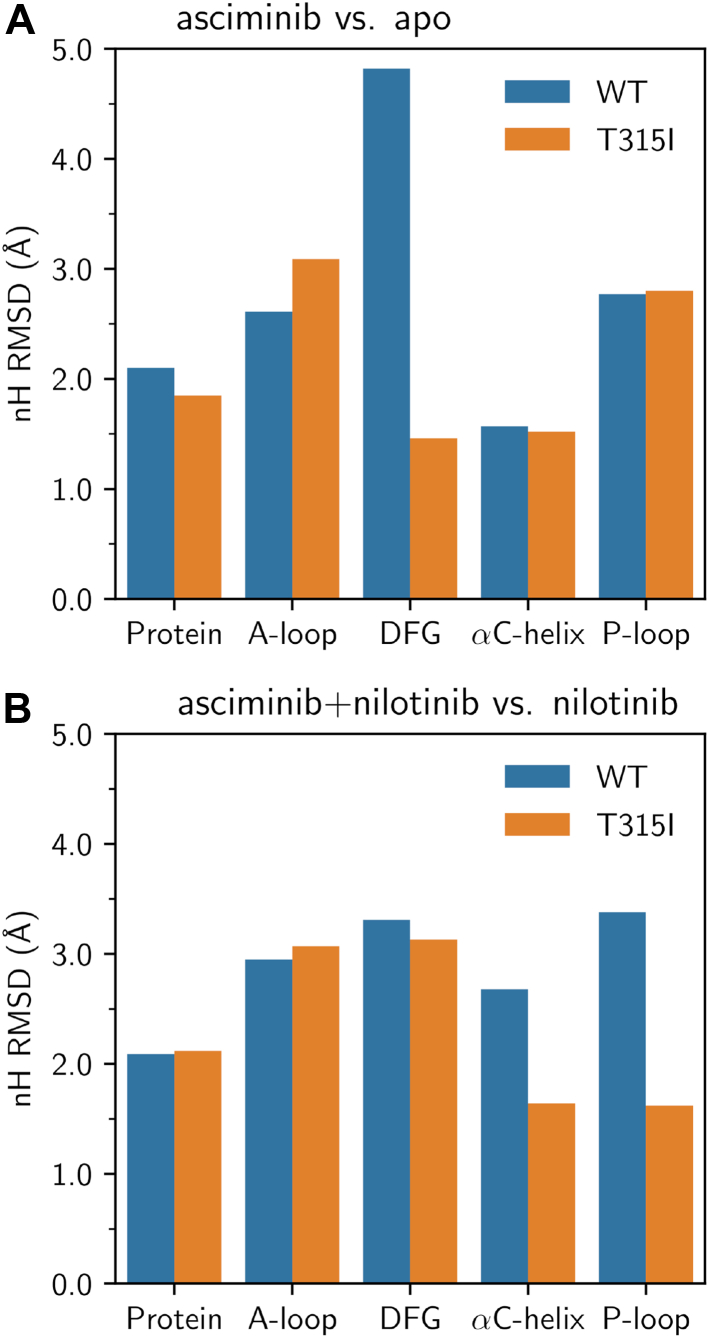
**RMSD values in the nonhydrogen (nH) atoms for the cluster1 of the wildtype (WT) and T315I-mutated Abl1 kinase systems.** Panel ***A*** shows the RMSD values of the asciminib-bound system relative to the apo protein, and panel ***B*** displays the RMSD values of the asciminib+nilotinib bound system relative to the nilotinib-bound protein. Before each RMSD calculation, structures were fitted using all the nH atoms of the protein.

Four MD trajectories of 100 ns were run for the unbound protein, protein–asciminib, protein–nilotinib, and protein–asciminib+nilotinib complexes for both the WT Abl1 kinase and the T315I mutant. Subsequently, a two-step clustering analysis (32), based on the nonhydrogen (nH) atoms of the protein, was performed on all the MD simulations as detailed in the Experimental Procedures section. As can be noted from the cluster populations provided in the Table S1 of the SI, the populations of the two predominant clusters, cluster1 and cluster2, constitute 50 to 78% and 10 to 28% of the total population, respectively, and the cumulative population of the two clusters covers 70 to 88% of the configurations. Thus, these clusters were considered to be representative of the system at hand, and the structure with largest number of neighbors in each cluster was considered for further analysis. For analyzing the changes in conformational dynamics of the inactive state of the protein upon the binding of asciminib, the nH root mean squared deviation (RMSD) using cluster1 of the asciminib-bound protein relative to the unbound protein and the asciminib+nilotinib bound protein relative to the nilotinib-bound protein are shown in Figure 4, *A* and *B*, respectively (see Fig. S3 of the SI for a similar analysis of cluster2).

**Figure 5:**
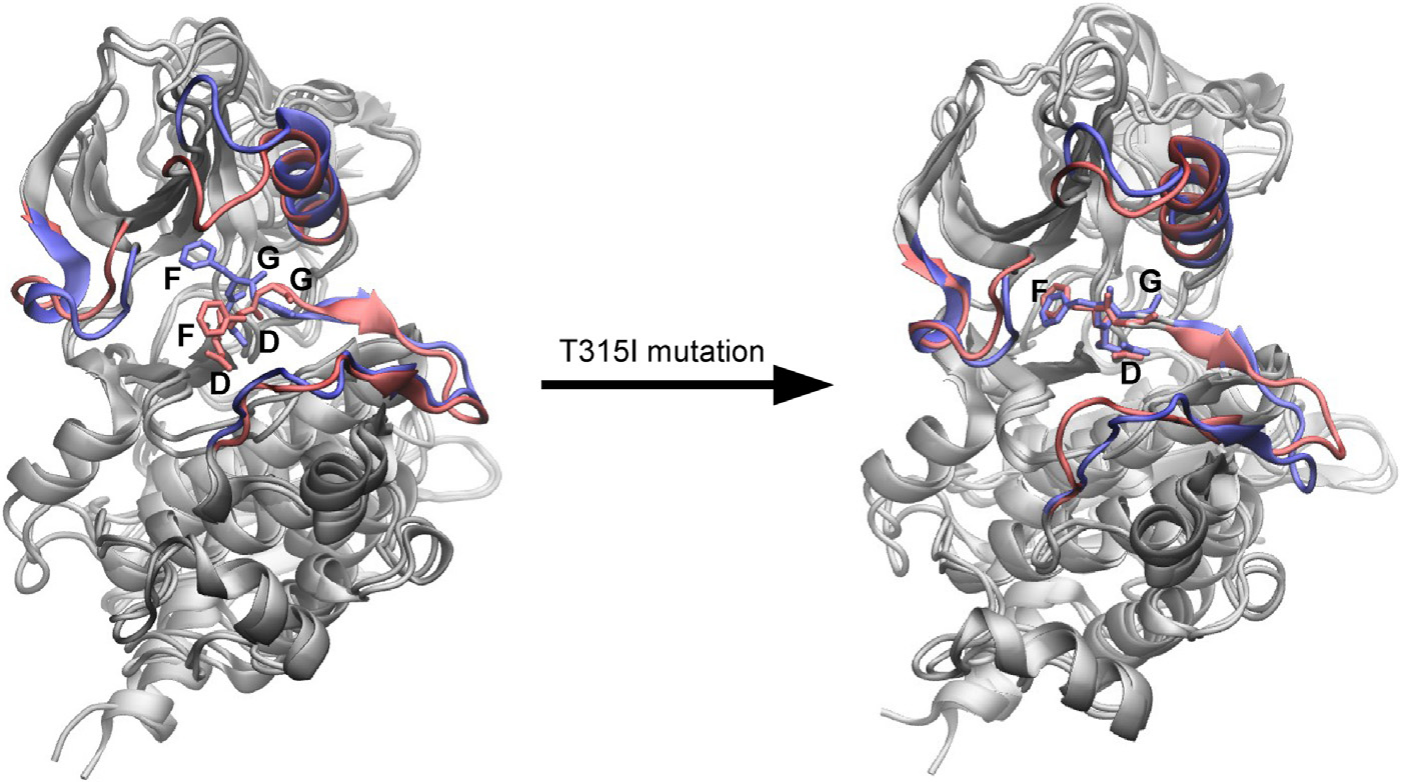
**Superposition of the asciminib bound (*red*) and the apo (*blue*) protein central structures of cluster1 for the wildtype and T315I-mutated systems.** The A-loop, the DFG-motif, the αC-helix, and the loop connecting it with the adjacent β-strand, and the P-loop are highlighted. Note the changes in the conformation of the DFG motif upon the T315I mutation. Asciminib is not shown.

Comparing the asciminib-bound protein with the unbound protein for the WT system, it can be seen from Figures 4*A* and 5 that the binding of asciminib triggers larger structural changes in the DFG motif compared to those in the A-loop, C-helix, and the P-loop. Specifically, the RMSD values of the A-loop, DFG motif, C-helix, and P-loop are 2.6, 4.8, 1.6, and 2.8 Å, respectively, while the full-protein RMSD is 2.1 Å. However, upon the T315I mutation, the structural changes in the DFG motif due to the binding of asciminib were substantially reduced (by 3.5 Å; Figs. 4*A* and 5). In contrast, comparing the asciminib+nilotinib–bound protein relative to nilotinib-bound protein, the RMSD values for the DFG motif (3.1–3.3 Å) were similar in both the WT system and the T315I mutant (Figs. 4*B* and S4 of the SI) but differed by 1.0 to 1.8 Å between the two for the C-helix and P-loop elements. Overall, our MD simulations suggest that the binding of asciminib triggers changes in the conformational dynamics of the inactive state of the protein, albeit these provide no significant insights on whether these structural changes promote or inhibit the activation.

#### Metadynamics simulations clearly demonstrate that asciminib hinders the activation of Abl1

**Figure 6:**
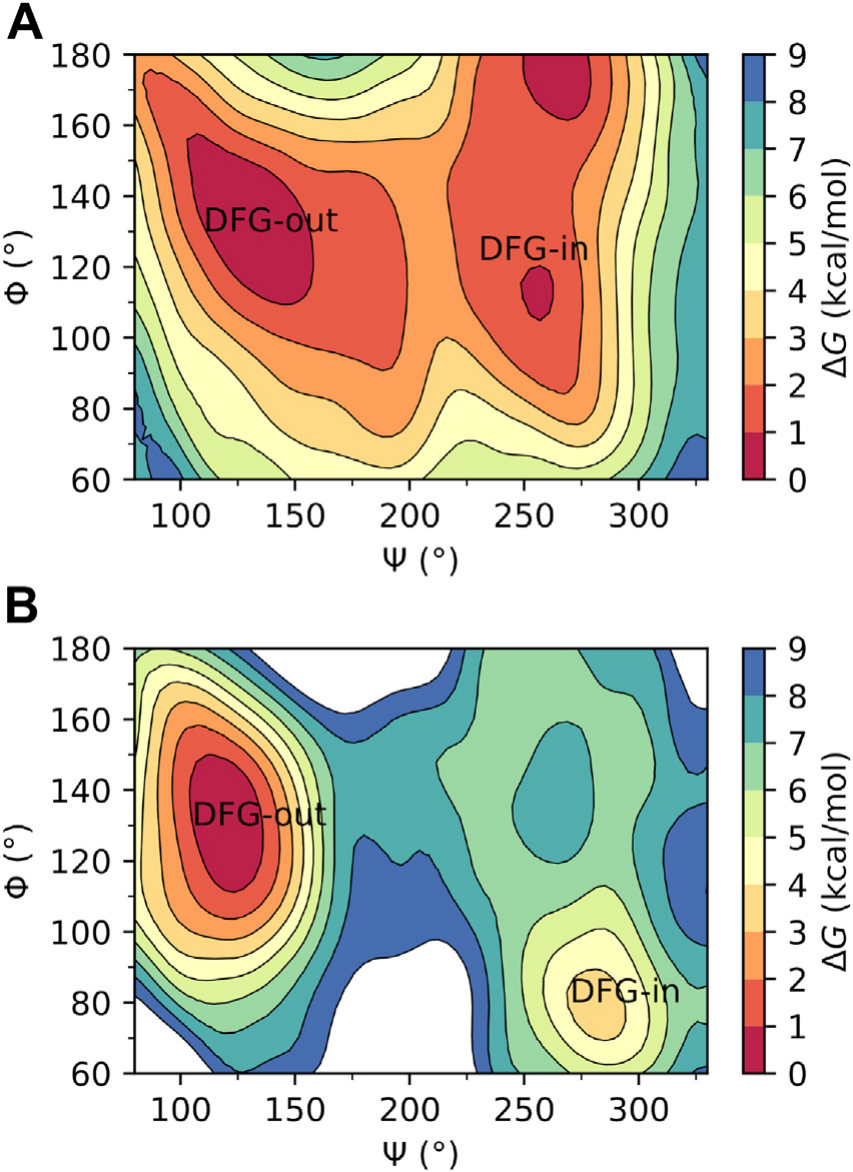
**Free-energy surfaces for the inactive (DFG-out) → active (DFG-in) transition of the Abl1 kinase as a function of the Ramachandran angles Ψ and Φ.** Panels ***A*** and ***B*** correspond to the unbound and asciminb-bound proteins, respectively.

**Figure 7:**
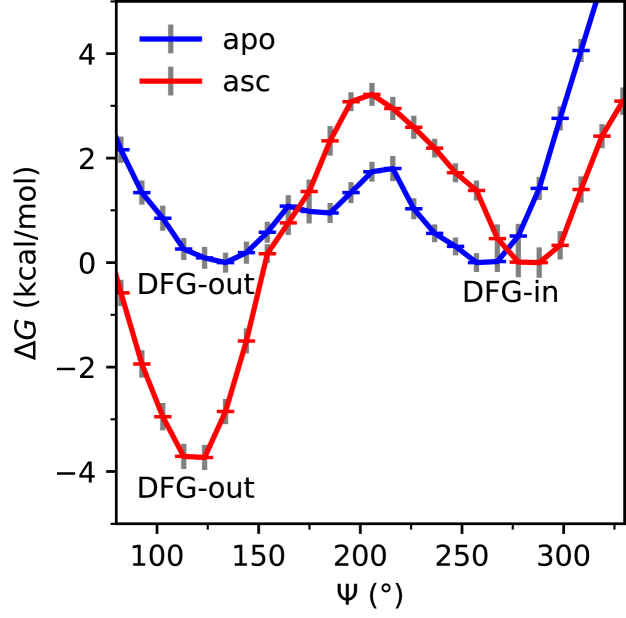
**Free-energy path for the inactive (DFG-out) → active (DFG-in) transition of the unbound (apo) and asciminib bound (asc) Abl1 kinase as a function of the Ramachandran angle Ψ**. Standard errors in free energies are shown as *gray vertical bars*. The free-energy of the DFG-in state was considered to be zero for both the systems.

To obtain quantitative insights into how the structural changes triggered by the binding of asciminib translate to changes in relative energies of the inactive DFG-out and active DFG-in states and the kinetics of activation, the DFG-flip of unbound as well as asciminib-bound WT protein systems were modeled by performing well-tempered metadynamics (wT-metaD) simulations using the Ramachandran angles Ψ and Φ (see Fig. S5 of the SI) and as collective variables. Free energy surfaces of the DFG-flip as a function of angles Ψ and Φ are shown in Figure 6, and the corresponding one-dimensional free energy projection on Ψ generated by integrating out Φ is shown in Figure 7.

As can be seen from Figure 6, the activation process involves larger changes in Ψ (125–140°) compared to those in Φ (20–50°) for both the unbound and asciminib-bound systems. Furthermore, in the case of unbound protein, the inactive and active states are equienergetic, which is in good agreement with previous computational studies of Abl1 kinase (33, 34). However, upon the binding of asciminib, the inactive state is stabilized by 3.7 ± 0.5 kcal/mol relative to the active state shifting the equilibrium distribution significantly toward the inactive state (see Fig. 7). As nilotinib binds the inactive state, a shift in equilibrium toward this state makes it more accessible for the binding. Furthermore, the free-energy barriers for the activation, calculated as 7.0 ± 0.5 and 1.8 ± 0.5 kcal/mol, respectively, for the asciminib-bound and unbound protein systems suggest that binding of asciminib hinders the activation. It is notable that although the estimated DFG-flip barrier of 1.8 kcal/mol for the unbound Abl1 kinase protein in this work seems to differ from some previous estimates of ∼7 kcal/mol (33, 35), it agrees well with the corresponding estimate by Lovera *et al.* (2.0 ± 1.0 kcal/mol) (36). This deviation can be understood in terms of the conformational variability in the set of inactive states in terms of changes in structural elements other than the DFG motif such as the A-loop, αC-helix, and P-loop (8, 37, 38). On the other hand, it is notable that while the previous studies considered only the kinase domain of the protein (33, 35, 36), the present study incorporates also the regulatory domains, albeit this difference does not explain the observed deviation of ∼5 kcal/mol between the barrier estimates of Lovera *et al.* (36) and other previous studies (33, 35). It is likely that rapid interconversion occurs between two distinct inactive conformations (37) that differ in the secondary structural elements noted above.

To further corroborate our findings and test the robustness of the simulation methodology, a complementary wT-metaD simulation was performed to model the DFG flip of the apo protein using a smaller bias factor (see the Experimental Procedures section). The results of this simulation, shown in Figs. S6 and S7 of the SI, confirm the finding that the inactive and active states have nearly identical energies and yield a DFG flip barrier (2.5 kcal/mol) that is in good agreement with the one obtained with a larger bias factor. Overall, wT-metaD simulations demonstrate that binding of asciminib skews the equilibrium toward the inactive state and raises the free-energy barrier of activation.

### Quantum chemical calculations reveal that the binding affinity of nilotinib is increased in the presence of asciminib

To investigate whether the presence of asciminib modulates the binding affinity of nilotinib in both WT Abl1 kinase and the T315I mutant, quantum chemical calculations using density functional theory (DFT) were performed on model systems of asciminib+nilotinib and nilotinib-bound Abl1 kinase complexes. Starting from the central structures of cluster1 and cluster2 and extracting only those residues that form hydrogen bonds with nilotinib in the ATP-binding pocket (following the protocol developed in our recent work (39)), the calculations were carried out at the M06/def2-TZVP//M06/def2-SV(P) level of theory (40). The results that are presented in Table 2 for cluster1 (see Table S2 of the SI for cluster2 results) show that asciminib increases the binding affinity (*i.e.*, reduces ΔG_b_^nil^) of nilotinib by 2.7 kcal/mol and 6.7 kcal/mol for the WT Abl1 kinase and T315I mutant, respectively. These results agree well with the observation from the cell line experiments that inhibition activity of nilotinib is enhanced by the co-binding of asciminib (Fig. 2*B*) and complements the finding from the wT-metaD simulations that asciminib facilitates binding of nilotinib by making the inactive state more accessible (Fig. 7). Furthermore, as can be seen from Table 2, the computed binding free energies of nilotinib are in good agreement with the previously reported values (26, 41, 42).

**Table 2:** Binding free energies (kcal/mol) of nilotinib (ΔG_b_^nil^), changes in ΔG_b_^nil^ upon the binding of asciminib (ΔΔG_b_^nil^), and changes in ΔG_b_^nil^ upon the T315I mutation (ΔΔG_b_^nil^(T315I)) for the central structures of the cluster1 (experimental ΔG_b_^nil^ (Exp.) values given for comparative purposes)

System	Drug(s)	Residues	ΔG_b_^nil^	ΔΔG_b_^nil^	ΔG_b_^nil^(T315I)
Wildtype	Nil	Glu^286^, Thr^315^, Met^318^	−12.6	–	–
Exp.	Nil [26, 41, 42]	–	[ −11.5, −10.7]	–	–
	Nil+asc	Glu^286^, Thr^315^, Met^318^	−15.3	−2.7	–
T315I	Nil	Glu^286^, Met^318^, Asp^381^	−8.2	–	+4.4
Exp.	Nil [42]	–	−8.4	–	–
	Nil+asc	Glu^286^, Val^289^, Met^318^, Asp^381^	−14.9	−6.7	+0.4

Interestingly, while the binding affinity of nilotinib (in the absence of asciminib) decreases by 4.4 kcal/mol upon the T315I mutation, the decrease is almost nullified (only 0.4 kcal/mol) by the presence of asciminib as can be noted from Table 2. Such allosteric enhancement of binding affinity of nilotinib by asciminib suggests that the combination alleviates resistance against the T315I mutant and restores the inhibition activity of nilotinib. This result is consistent with a recent finding that nilotinib in the presence of asciminib suppresses the emergence of resistance in CML cells (14, 17). Thus, combination therapy of asciminib+nilotinib might be a promising strategy in the treatment of patients diagnosed with T315I-mutated CML.

**Figure 8:**
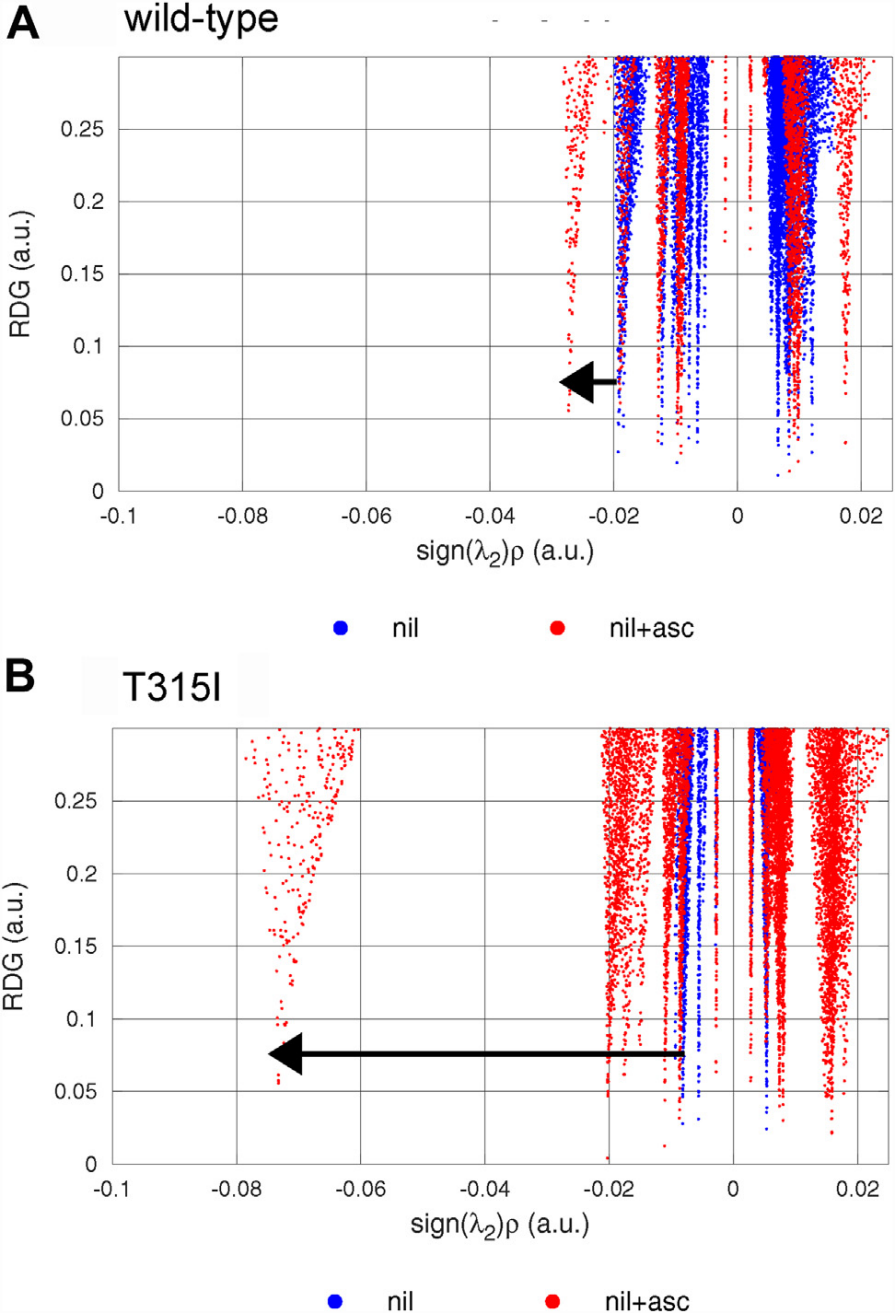
**Noncovalent interaction plots of the Glu**^**286**^**residue in the presence (*red dots*) and absence of asciminib (*blue dots*) for cluster1 of the wildtype and T315I-mutated Abl1 kinases.** In the depicted gradient isosurfaces, *low* reduced electron density gradients (RDGs) and *low* electron densities (ρ) characterize noncovalent interactions. The magnitude of ρ represents the strength of a given interaction, and the negative (positive) sign of the eigenvalue (λ_2_) of the Hessian of electron density denotes attractive (repulsive) interaction. *Arrows* indicate the direction of change in the strength of the hydrogen bonding upon the binding of asciminib (a large negative value suggests a stronger interaction). Abl1, Abelson tyrosine kinase.

Finally, to identify the interactions that contribute to the allosteric enhancement in binding affinity of nilotinib by asciminib, noncovalent interaction (NCI) analysis (43) was performed on protein residues that interact with nilotinib (see Fig. S8 of the SI) in the presence and absence of asciminib. Noting in Figure 8 that the negative (positive) side of the X-axis represent attractive (repulsive) interactions, and the magnitude of the electron density (ρ) indicates associated strength, the results presented for the Glu^286^ residue suggest that the binding of asciminib improves the strength of hydrogen bonding between the N–H hydrogen of nilotinib and the carboxylic oxygen atoms of the Glu^286^ residue. This can also be inferred from the decrease in the N–O donor–acceptor (D–A) distances from 3.7 to 3.1 Å (see Table S3 of the SI). The improvement is particularly significant in the case of T315I mutant, wherein the peak corresponding to this interaction is displaced to the left (becomes favorable) in the NCI analysis (Fig. 8*B*) and the D–A distance decreases by ∼1.0 Å (Table S3) upon the binding of asciminib. Thus, the Glu^286^ residue situated in the αC-helix of the protein seems critical for restoring the lost inhibition activity of nilotinib against the T315I mutation, and any mutation at this position might negatively impact the inhibition activity. Turning to the NCI plots for other residues shown in Fig. S9 of the SI and the corresponding D–A distances in Table S3 of the SI (see Fig. S10 and Table S4 of the SI for cluster2 results), it can be noted that Thr^315^ residue also contributes to the increased binding affinity of nilotinib in the presence of asciminib for the WT system. Although this interaction is lost upon the T315I mutation, it is compensated by the stronger interaction of nilotinib with Glu^286^.

## Conclusions

We have presented a combined experimental and computational investigation exploring the effectiveness of asciminib+nilotinib and asciminib+dasatinib combination therapies in inhibition of the BCR-Abl1 kinase activity and the underlying molecular mechanisms. Performing experiments on CML K562 and KCL-22 cell lines, we have observed that the binding of asciminib enhances the inhibition activity of nilotinib by decreasing its IC_50_ value by up to half an order of magnitude relative to that in its monotherapy. By carrying out MD and wT-metaD simulations, it was found that one reason for such enhancement is that asciminib triggers structural changes in the ATP-binding domain of the inactive state of the protein, thereby making the activation process thermodynamically unfavorable due to increased endergonicity of the reaction by ∼4 kcal/mol. Furthermore, through quantum chemical calculations, we have demonstrated that asciminib co-binding decreases the binding free energy of nilotinib by ∼3 kcal/mol for the WT and ∼7 kcal/mol for the T315I-mutated Abl1 kinase systems by improving the strength of hydrogen bonding between the Glu^286^ residue of the protein and nilotinib. Overall, our findings suggest the possibility to reduce the dose of nilotinib in case of dose-related toxicities and alleviate its resistance against the T315I mutation by combining it with asciminib.

## Experimental procedures

### Cell line experiments

#### K562 cells

K562 cells were thawed and grown in media consisting of RPMI 1640 medium (with GlutaMAX, Gibco), 10% FBS (fetal bovine serum, Gibco), and 1% antibiotics (Penicillin and streptomycin, Gibco). Asciminib (MedChemTronica), nilotinib (SignalChem), and dasatinib (SignalChem) were purchased and dissolved in DMSO (SigmaAldrich).

1.4 × 10^4^ K562 cells were seeded per well in a 96-well cell culture plate with different concentrations of inhibitors in 100 μl medium. Each concentration was run in triplicate. For asciminib, the highest concentration used was 12.8 nM, which was then halved in each subsequent well to a concentration of 0.00625 nM in the 12th well. Adapting the same procedure, nilotinib and dasatinib concentrations were varied between 200 to 0.0977 nM and 64 to 0.0313 nM, respectively. The plates were kept in an incubator for 48 h. To assess cellular proliferation, 20 μl of MTS (methanethiosulfonate) reagent was added, and the plates were incubated again for 2 h. Absorbances were then measured at 490 nm, and the results were used to construct dose–response curves. The IC_50_ values of the inhibitors were estimated using nonlinear regression analysis with the two-parameter Hill equation model in GraphPad Prism9 software.

The same procedure was then employed for drug combinations of asciminib+nilotinib and asciminib+dasatinib at concentration ratios of 1:4.5 and 2.7:1, respectively. The asciminib:nilotinib ratio was chosen to be close to the previously employed ratio of 1:5 that yielded encouraging results in terms of inhibition of drug resistance (17), whereas the asciminib:dasatinib ratio was altered from the 4:1 value employed in our recent study that resulted in a predominance of antagonism between the drugs (20). For the asciminib+nilotinib combination, concentration ranges for asciminib and nilotinib were 26.4 to 0.013 nM and 118.4 to 0.058 nM, respectively. For the asciminib+dasatinib combination, concentration ranges for asciminib and dasatinib were 52.8 to 0.026 nM and 19.68 to 0.0096 nM, respectively.

CI values for the drug combinations were calculated using Loewe's model of drug synergy as implemented in the synergy package of Python (44). In the implementation of this model, single drug responses were fitted using four parameter Hill equation by performing nonlinear regression analysis to obtain the optimal parameters. This contrasts with the median-effect equation model that log transforms the dose–response data and uses linear regression to generate the fitted parameters (44).

#### KCL-22 cells

KCL-22 cells were maintained in suspension culture using the same protocol as described above for K562 cells. Additionally, resistant cell lines (KCL-22NR) were developed by growing KCL-22 cells in the cell culture medium that contains 10 nM nilotinib. Briefly, exponentially growing cells were collected and adjusted to 1 × 10^5^ cells/ml. 1 ml of cell suspension per well was seeded in 24-well plates with 10 nM nilotinib. Cells treated with the same concentration of DMSO (0.1% (vol/vol)) were set as control. Media and compounds were replenished every 4 days. Cell numbers were monitored every other day. This process was repeated until the culture time was 7 weeks to get stable drug resistant cell lines.

For both the KCL-22 and KCL-22NR cell lines, cells were seeded into 96-well culture plates with the concentration of 10,000 cells/well and with different concentrations (ranging from 320 to 0.15625 nM by halving at each subsequent well) of either asciminib or nilotinib. Each concentration was run in duplicate, and the plates were incubated for 48 h. Cellular proliferation was assessed using MTS reagent, and the IC_50_ values were obtained as described above.

For the combination assay, cells were plated in triplicate in 96-well plates. Concentration of asciminib was fixed at its IC_50_ value against KCL-22 cells, while concentration of nilotinib was varied from 320 to 0.15625 nM. DMSO concentration was kept constant and did not exceed 0.1% of the total volume. Cell proliferation was determined with the same protocol as described above.

### Molecular dynamics and metadynamics simulations

All MD simulations were performed with the Gromacs program, v2020.1 (45, 46), and wT-metaD (47) simulations were performed using the PLUMED (48, 49) version of Gromacs, v2019.5-plumed. The CHARMM36 (50) force field was employed for solute atoms, and the TIP3P (51) model was used for water molecules. A cutoff distance of 1.2 nm was used for computing van der Waals and Coulomb interactions. The long-range electrostatic interactions were treated with the PME method (52, 53). The LINCS (54) and the SETTLE (55) algorithms were employed to constrain bonds having hydrogen atoms and rigid water molecules, respectively. The simulations were carried out with a time step of 2 fs in the NPT ensemble at T = 300 K, using the velocity rescaling thermostat (56) (time constant of 0.1 ps), and at *p* = 1 bar, by using the Berendsen barostat (57) (time constant of 2.5 ps) for the positional restraints and equilibration simulations, and by the Parrinello-Rahman barostat (58) (time constant of 2.5 ps) for the production MD runs.

#### MD simulations

For both the WT and T315I-mutated Abl1kinase systems, four different systems were considered: apo Abl1, Abl1+asciminib, Abl1+nilotinib, and Abl1+asciminib+nilotinib. The starting structure for the preparation of all the input files was the Abl1+asciminib+nilotinib complex obtained from Protein Data Bank (PDB) (59): PDB-ID 5MO4 containing the T315I and D363N mutations (14). Coordinates from the PDB structure 1OPL (60), a structure of the autoinhibited Abl1 with an inhibitor (PD166326) and myristoyl pocket, were used to complete the missing parts of the 5MO4 structure using the Chimera program (61). The myristic acid in the myristoyl pocket and the PD166326 inhibitor were not included in the starting structures, but the D363N mutation of the 5MO4 structure was retained as we did not expect its effects to influence the overall dynamics of the protein.

Prior to the MD simulations, energy minimization was performed on all the structures either up to 5000 steps or until a maximal force of 100 kJ mol^-1^ nm^-1^ was reached. Subsequently, a short 50 ps MD simulation was performed with positional restraints on all the nH atoms of the protein in order to relax the water molecules and ions surrounding it. For the system to fully relax, a 5 ns equilibration simulation was performed after removing the restraints. Production MD simulations of 100 ns were then carried out at constant pressure (1 bar) and temperature (300 K) by running four different trajectories for each system. Coordinates and energies were saved every 2 ps.

A two-step clustering analysis based on the nH atoms of the protein, using the algorithm developed by Daura *et al.* (62), was performed on all the MD simulations. In the first step, a RMSD cut-off of 0.15 nm was used for clustering each MD trajectory. Next, the central structures of all clusters were combined into a new trajectory, and a second clustering step was performed with a RMSD cut-off of 0.25 nm.

#### Metadynamics simulations

In metadynamics simulations (47, 63, 64, 65), a history-dependent Gaussian bias potential as a function of a few collective variables is added to the Hamiltonian of the system that pushes the system out of the energy well and facilitates an efficient exploration of the free-energy space. In wT-metaD simulations (47), the Gaussian height is tempered during the simulation to avoid overfilling of the energy wells and to enable smooth convergence of the final bias potential. In the present work, wT-metaD simulations were run for 125 ns for the unbound WT Abl1 kinase and for 140 ns for the corresponding asciminib-bound system. Gaussians of width 0.2 radian were deposited every 500 steps with the initial height set to 0.5 kJ/mol and using a bias factor of 15 (a bias factor of 8 and a Gaussian width of 0.1 radian were used for one of the simulations to validate the results).

One-dimensional projections of the free-energy surfaces on Ψ were generated by splitting the full configuration space of into 72 bins of equal width and choosing the value of Φ that yielded the lowest free energy within the each bin. Standard errors in free energies were estimated using an umbrella sampling-like approach to obtain the unbiasing weights and a block averaging procedure to remove time correlations in the data (35, 66).

### Quantum chemical calculations

#### Binding free energies

Model systems for calculating binding free energies with DFT were prepared by considering only those residues that form hydrogen bonds (allowing for D–A distances of up to 4.1 Å, *i.e.*, considering even weak interactions) with nilotinib by using the PLIP website (67). Methyl groups were added on the peripheral atoms to satisfy the valence requirements. All the model systems were first optimized in gas phase at the M06/def2-SV(P) (40, 68) level of theory by freezing the heavy atoms. Subsequently, using the optimized geometries, single-point energy calculations were performed at M06/def2-TZVP (40, 68) level of theory using the SMD implicit solvation model (69) to describe the water solvent. The Gibbs binding free energy of nilotinib (Δ*G*_b_^nil^) was obtained as the difference between the free energy of the Abl1+nilotinib complex (G_Abl1-nil_) and the sum of free energies of the isolated nilotinib (G_nil_) and Abl1 (G_Abl1_) systems:

(1) $$\Delta G_{b}^{nil}=G_{Abl1-nil}-(G_{nil}+G_{Abl1})$$

All the geometry optimizations were performed using the NWChem program (70), version 6.8.1, and the singlepoint calculations were carried out with the GAMESS program (71, 72), version 2012 (R1).

#### NCIs analysis

NCI analysis uses properties of the electron density and the reduced electron density gradient to distinguish between different types of chemical interactions (43). NCIs are characterized by low electron density and low reduced density gradient values and can be visualized as gradient isosurfaces (43). Taking the optimized model systems from the DFT calculations, NCI analysis was carried out for each protein residue interacting with nilotinib using the NCI plot 4 program (73), and the results were plotted with the Octave program https://www.gnu.org/software/octave/doc/v5.2.0/.

## Data availability

All data are present in the Manuscript and the Supporting Information. The simulation trajectories can be made available upon request to the authors.

## Supporting information

This article contains supporting information.

## Conflict of interest

The authors declare that they have no conflicts of interest with the contents of this article.

## Supporting information

apo-DFG-flip

asc-DFG-flip
