# Supplementary material for: Allosteric enhancement of the BCR-Abl1 kinase inhibition activity of nilotinib by cobinding of asciminib
Source: J Biol Chem. 2022 Jul 6;298(8):102238. doi: 10.1016/j.jbc.2022.102238 (PMC9386466; doi:10.1016/j.jbc.2022.102238)
Supplement: Supporting information [file mmc3.pdf]

Supporting Information: Allosteric Enhancement of the BCR-Abl1 Kinase Inhibition Activity of Nilotinib by Co-Binding of Asciminib

Baswanth Oruganti[1],* Erik Lindahl[1], Jingmei Yang[1], Wahid Amiri, Rezwan Rahimullah, and Ran Friedman*

Department of Chemistry and Biomedical Sciences, Faculty of Health and Life Sciences, Linnæus University, 391 82 Kalmar, Sweden

E-mail: baswanth.oruganti@lnu.se; ran.friedman@lnu.se

Table S1: Populations (in percentages) of different clusters obtained from the two-step clustering analysis of the MD simulations of different systems.

system	drug(s)	cluster1	cluster2	cluster3	other clusters
wild-type	None	66	15	11	8
	asciminib	50	20	20	10
	nilotinib	59	16	13	12
	asciminib+nilotinib	78	10	9	3
T315I	None	74	11	6	9
	asciminib	58	28	6	8
	nilotinib	64	18	12	6
	asciminib+nilotinib	62	15	13	10

Comments. As can be noted from Table S1 above, cluster1 is the predominant cluster for all the systems. Specifically, for the asciminib+nilotinib bound and the nilotinib only Abl1 kinases, cluster1 constitutes 59–78% of the configurational space.

Table S2: Binding free energies (kcal/mol) of nilotinib ($\Delta G_{\rm b}{}^{\rm nil}$), changes in $\Delta G_{\rm b}{}^{\rm nil}$ upon the binding of asciminib ($\Delta\Delta G_{\rm b}{}^{\rm nil}$), and changes in $\Delta G_{\rm b}{}^{\rm nil}$ upon the T315I mutation ($\Delta\Delta G_{\rm b}{}^{\rm nil}$(T315I)) for the central structures of cluster2 (experimental $\Delta G_{\rm b}{}^{\rm nil}$ (Exp.) values were taken from references provided in the Table 2 of the manuscript).

system	drug(s)	residues	$\Delta G_{\rm b}^{\rm nil}$	$\Delta G_{\rm b}^{\rm nil}$	$\Delta G_{\rm b}^{\rm nil}$(T315I)
wild-type	nil	Glu^{286}, Thr^{315}, Met^{318}, Asp^{381}	−16.6	–	–
Exp.	nil	–	[−11.5, −10.7]	–	–
	asc+nil	Glu^{286}, Thr^{315}, Met^{318}, Asp^{381}, Leu^{384}	−13.3	+3.3	–
T315I	nil	Glu^{286}, Met^{318}, Val^{299}	−12.2	–	+4.4
Exp.	nil	–	−8.4	–	–
	asc+nil	Glu^{286}, Met^{318}, Asp^{381}	−14.2	−2.0	−0.9

Comments. For cluster2 of the wild-type asciminib+nilotinib (asc+nil) bound Abl1 kinase, the binding free energy of nilotinib (nil) is ∼3 kcal/mol higher than that for the nilotinib-bound protein. However, as can be noted from Table S1, cluster2 of the asciminib+nilotinib bound protein has a population of only 10% compared to the population of 78% for cluster1. Hence, the binding free energy of nilotinib for cluster2 is not representative.

Table S3: Donor-acceptor distances (Å) for the hydrogen bonds between the protein and nilotinib for the asciminib+nilotinib bound (asc+nil) and the nilotinib-bound (nil) systems for cluster1.

system	Glu^{286} (N—O)	Thr^{315} (N—O)	Met^{318} (N—N)	Asp^{381} (N—O)
wild-type				
nil	3.67	3.56	2.89	–
asc+nil	3.05	3.06	2.96	–
T315I				
nil	3.55	–	3.00	4.02
asc+nil	2.56	–	3.18	4.02

Table S4: Donor-acceptor distances (Å) for the hydrogen bonds between the protein and nilotinib for the asciminib+nilotinib bound (asc+nil) and the nilotinib-bound (nil) systems for cluster2.

system	Glu^{286} (N—O)	Thr^{315} (N—O)	Met^{318} (N—N)	Asp^{381} (N—O)	Leu^{384} (N—N)
wild-type					
nil	3.34	3.33	3.18	2.97	–
asc+nil	3.22	4.15	2.85	3.95	3.68
T315I					
nil	3.30	–	3.10	–	–
asc+nil	2.92	–	3.26	2.83	–

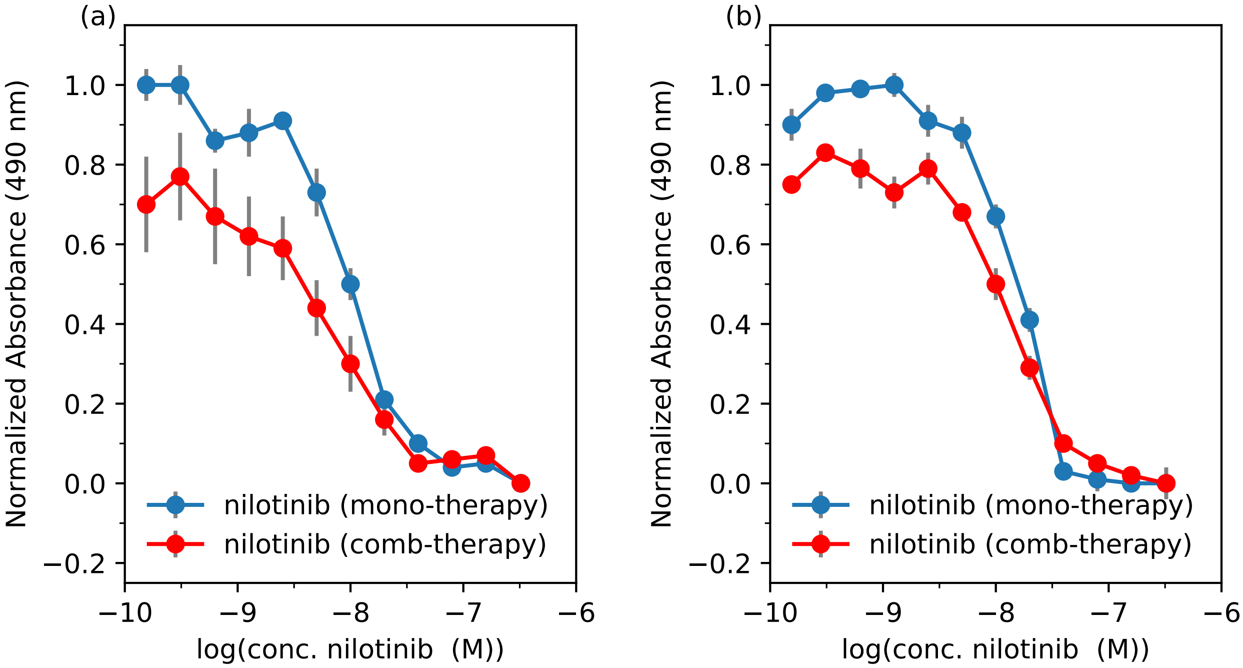

Figure S1: Dose-response curves for combination therapy of asciminib+nilotinib versus monotherapy of nilotinib against KCL-22 (a) and nilotinib-resistant KCL-22 (b) cells.

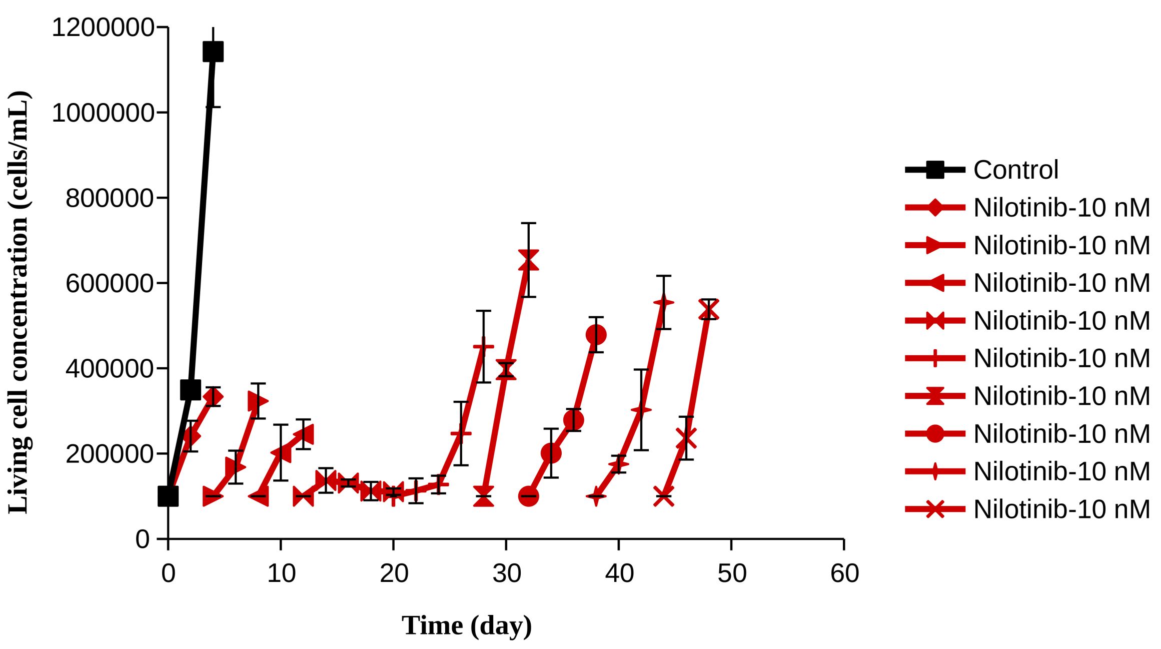

Figure S2: Changes in average cell concentrations as a function of time in the cell culture medium containing 10 nM nilotinib. Cells treated with the same concentration of DMSO (0.1% (vol/vol)) set as control. Standard deviations are shown as black vertical bars. The increase in cell concentrations after 20 days suggest development of nilotinib resistance.

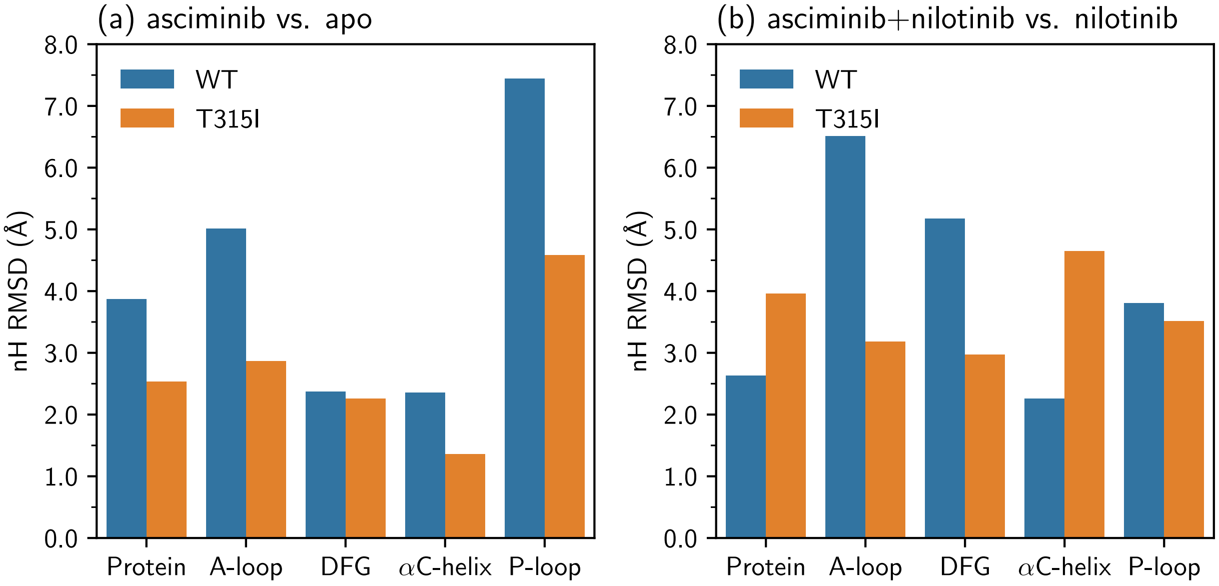

Figure S3: RMSD values in the non-hydrogen (nH) atoms of the asciminib bound versus the apo protein (a) and the asciminib+nilotinib bound versus the nilotinib-bound protein (b) for cluster2 of the wild-type (WT) and T315I-mutated Abl1 kinases.

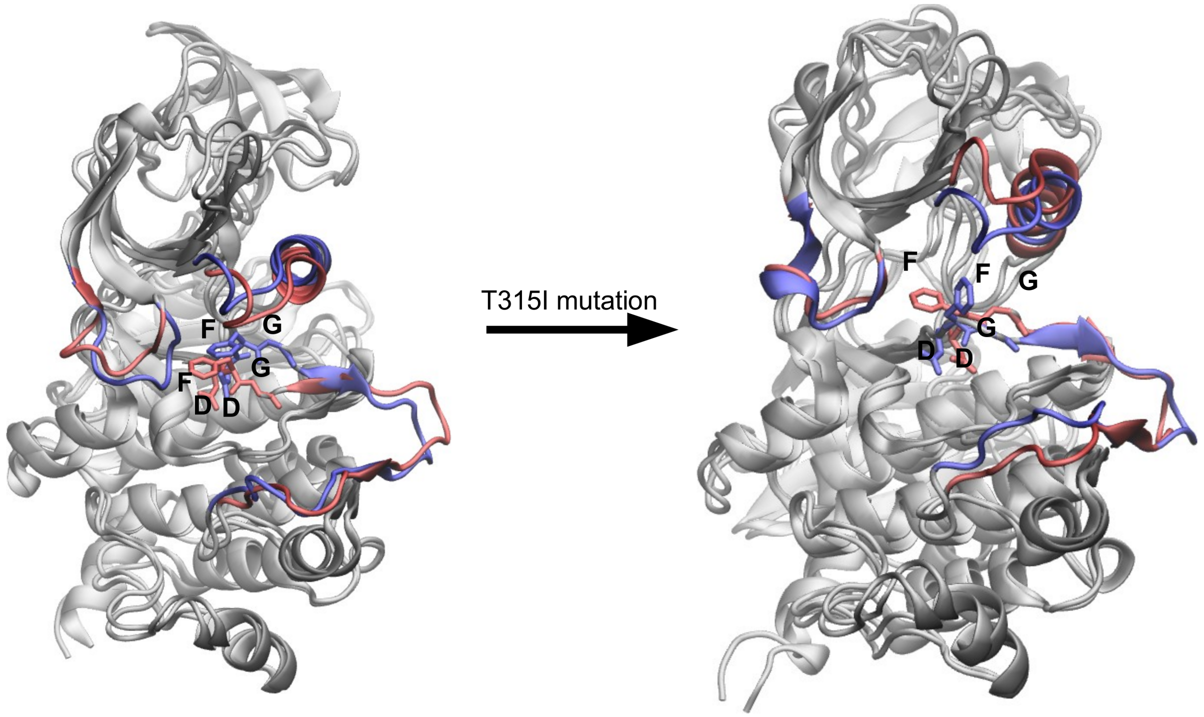

Figure S4: Superposition of the asciminib+nilotinib bound (red) and the nilotinib-bound (blue) protein central structures of cluster1 for the wild-type and T315I mutated Abl1 kinases. The A-loop, the DFG-motif, the αC-helix and the loop connecting it with the adjacent β-strand, and the P-loop are highlighted. Note the changes in the conformation of the DFG motif upon the T315I mutation. Asciminib and nilotinib are not shown.

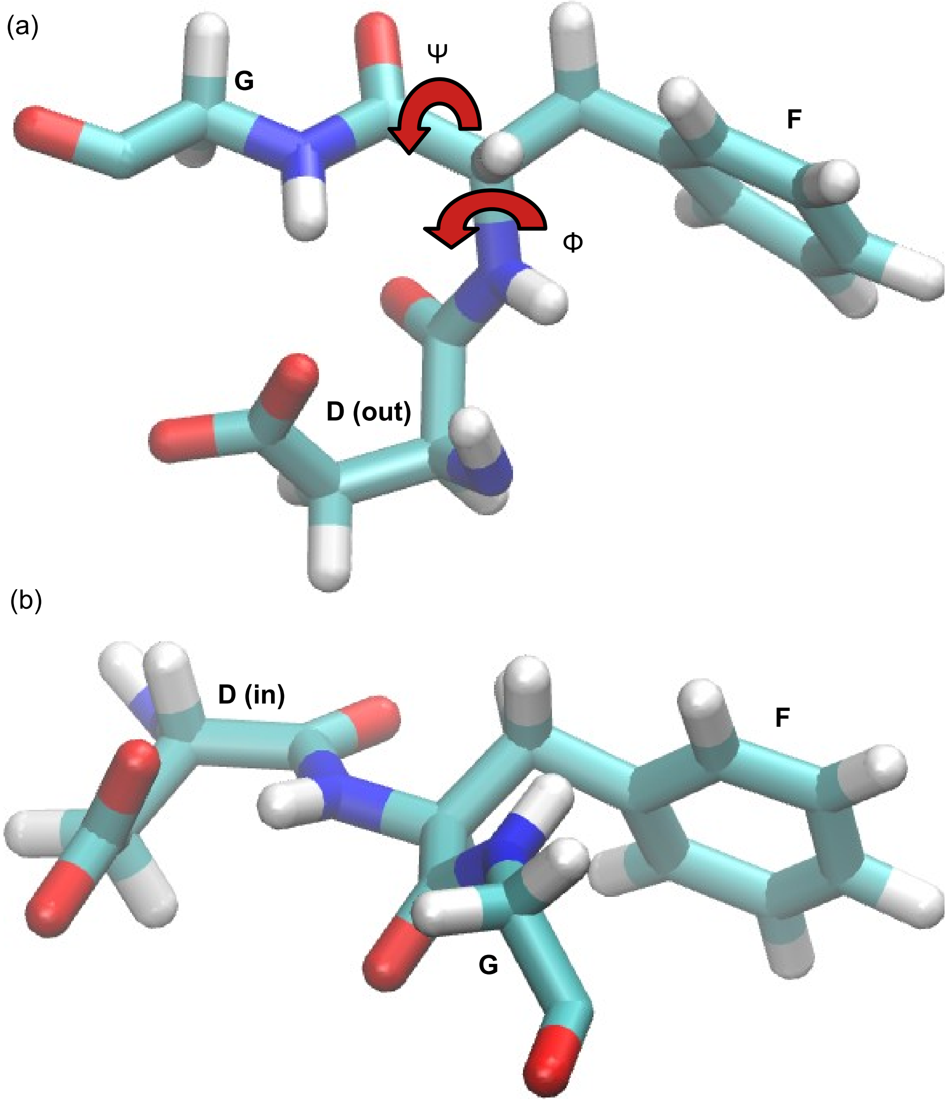

Figure S5: Structures of the DFG-out (a) and DFG-in (b) conformations. The Ramachandran angles Ψ and Φ are also shown.

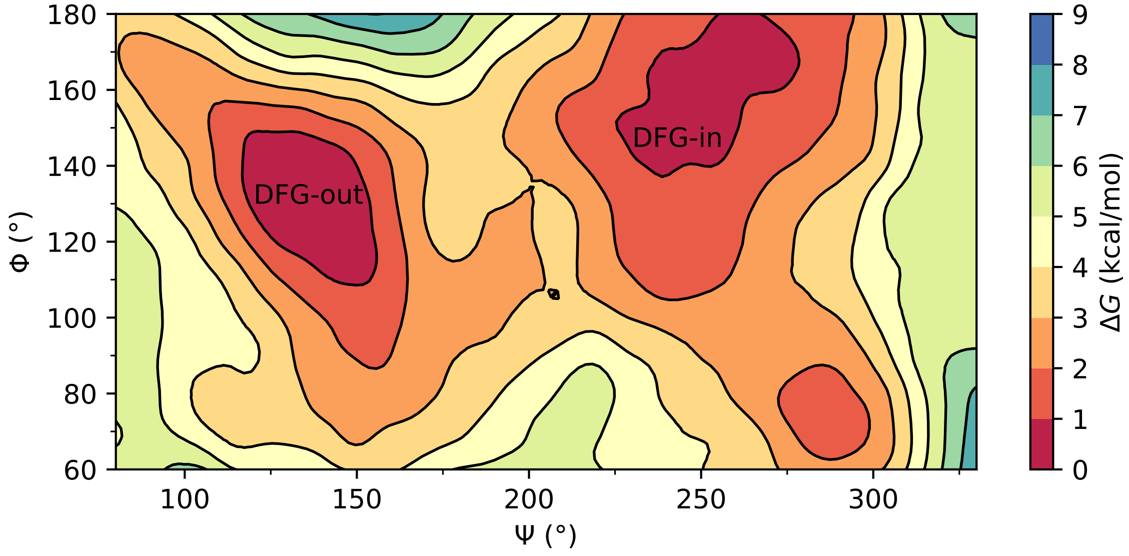

Figure S6: Free-energy surfaces for the inactive (DFG-out) → active (DFG-in) transition of the unbound Abl1 kinase as a function of the Ramachandran angles Ψ and Φ.

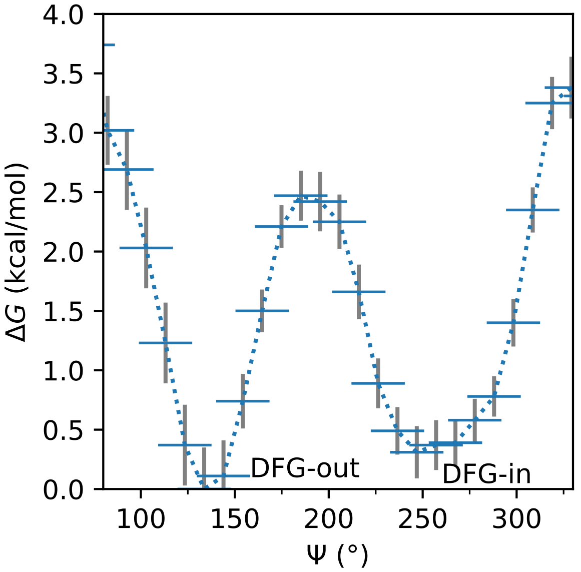

Figure S7: Free-energy path for the inactive (DFG-out) → active (DFG-in) transition of the unbound Abl1 kinase as a function of the Ramachandran angle Ψ. Standard errors in free energies are shown as grey vertical bars.

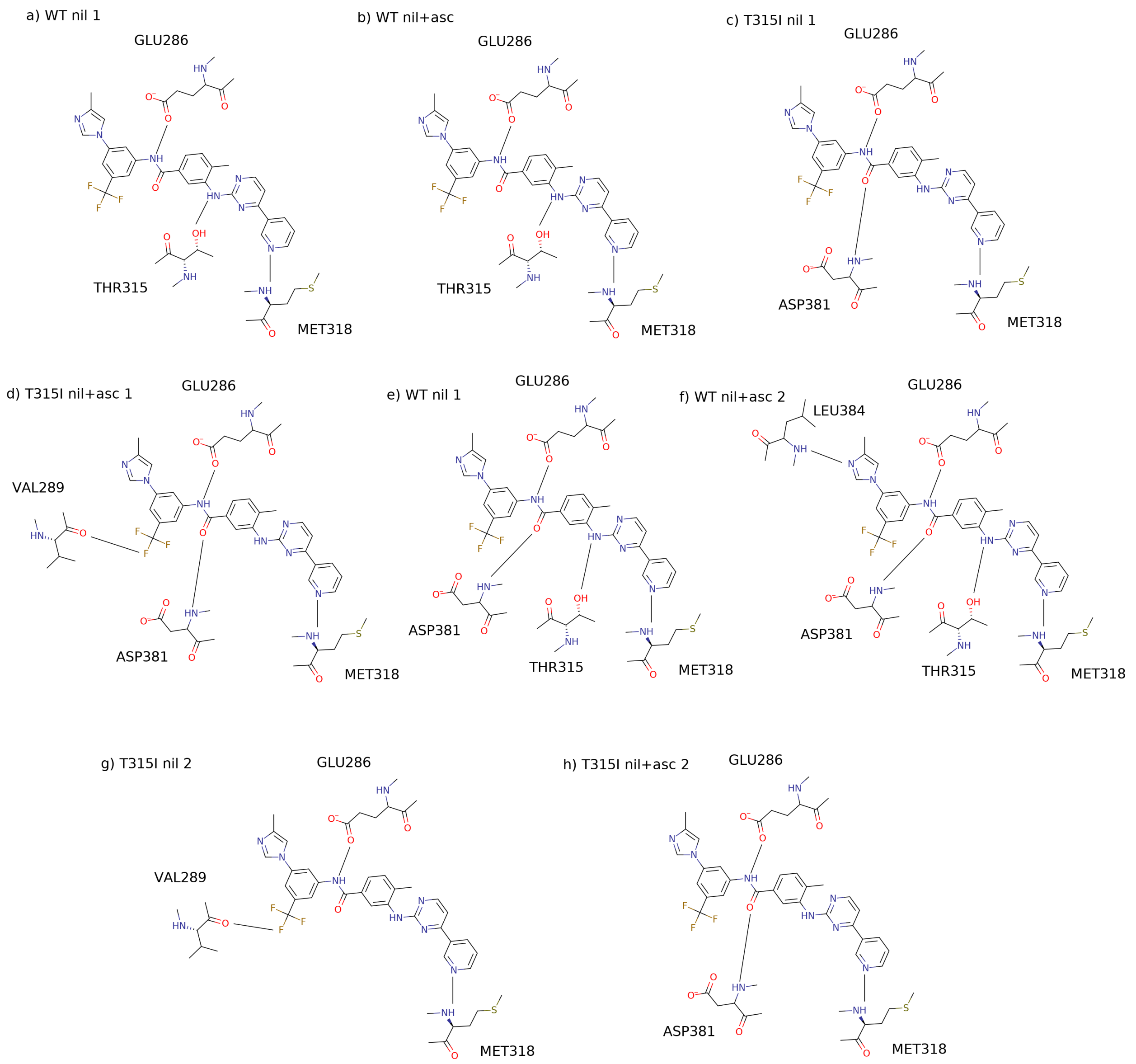

Figure S8: Structures of the model systems of the nilotinib binding site in asciminib+nilotinib bound (asc+nil) and nilotinib-bound (nil) wild-type (WT) and T315I-mutated Abl1 kinases considered for DFT calculations. 1 and 2 represent cluster1 and cluster2, respectively.

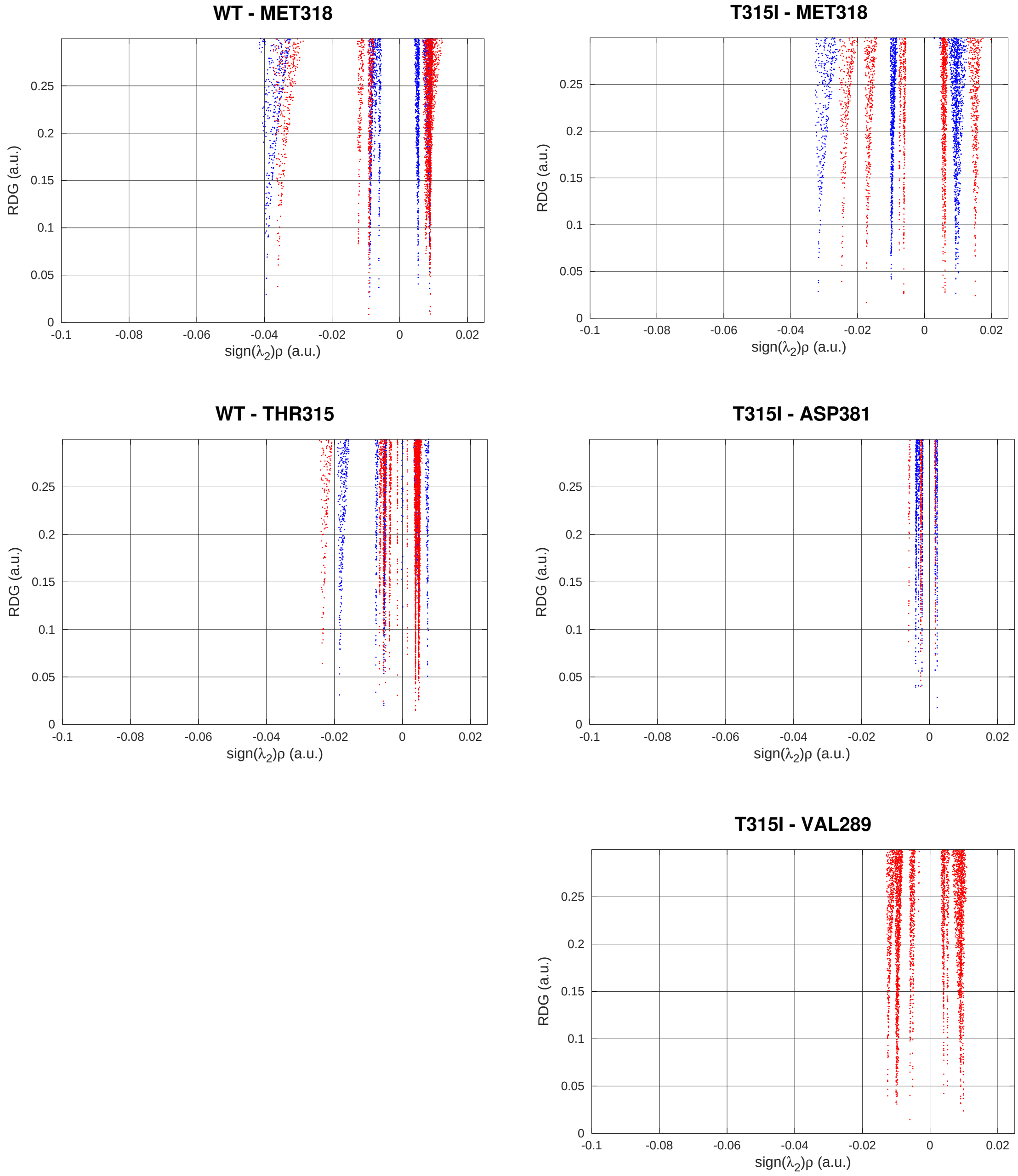

Figure S9: Non-covalent interaction plots in the presence (red dots) and absence of asciminib (blue dots) for the wild-type (WT) and T315I-mutated Abl1 kinases for cluster 1.

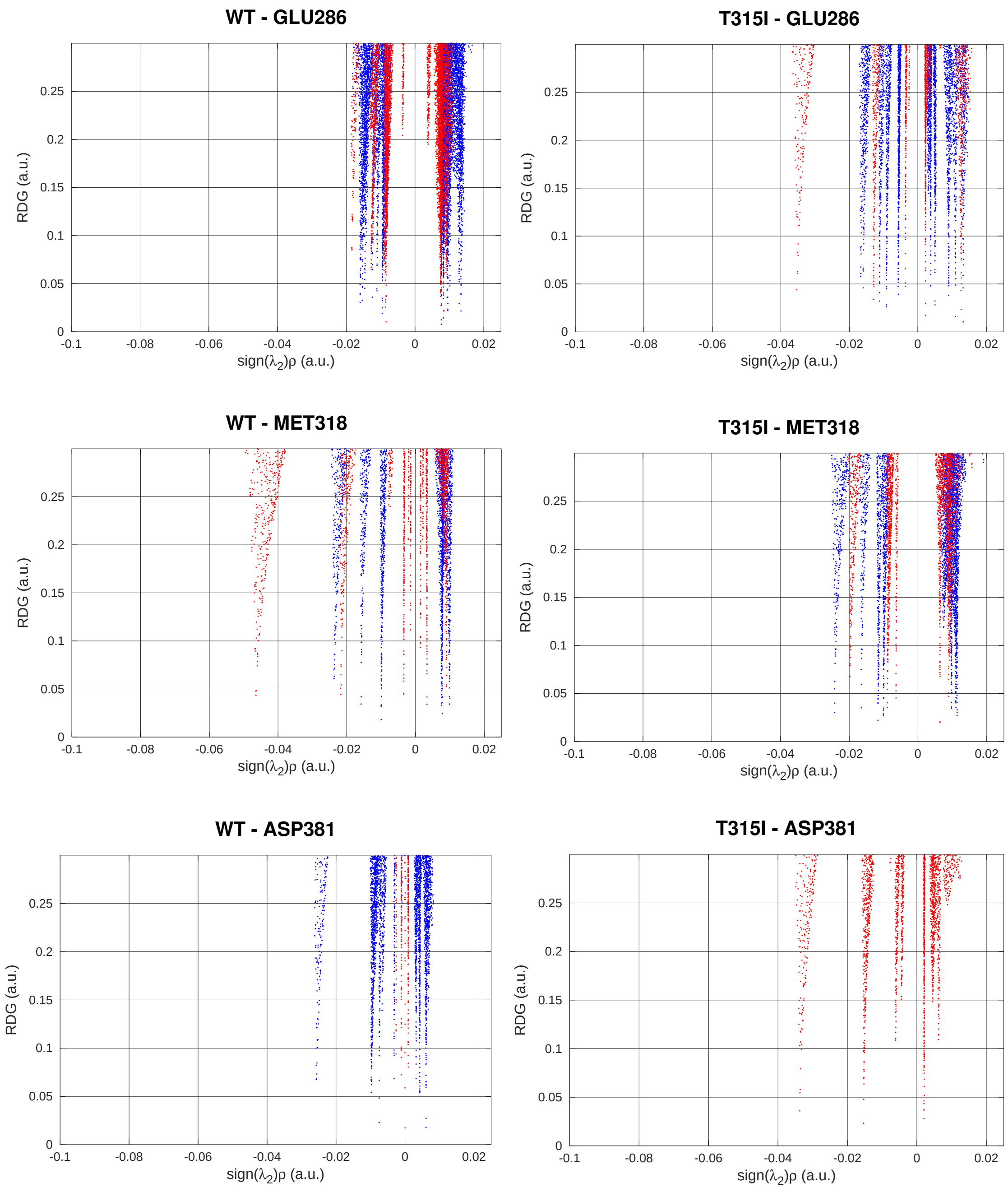

Figure S10: **continued to next page**

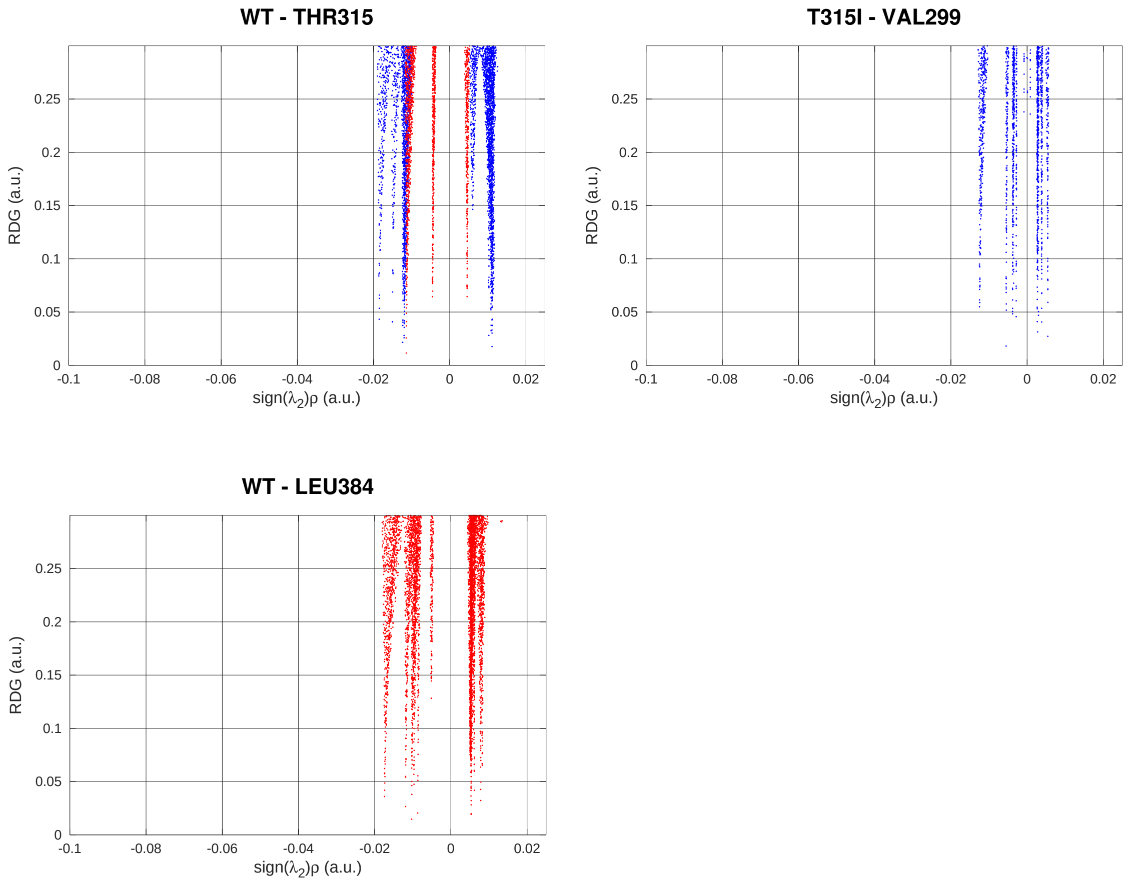

Figure S10: Non-covalent interaction plots in the presence (red dots) and absence of asciminib (blue dots) for the wild-type (WT) and T315I-mutated Abl1 kinases for cluster2.
